# Extra Cellular Matrix Derived Metabolite Regulates Angiogenesis by FasL Mediated Apoptosis

**DOI:** 10.1371/journal.pone.0080555

**Published:** 2013-12-04

**Authors:** Raj K. Verma, Venugopal Gunda, Smita C. Pawar, Yakkanti Akul Sudhakar

**Affiliations:** 1 Irma Lerma Rangel College of Pharmacy, Texas A&M Health Science Center, Kingsville, Texas, United States of America; 2 The Eppley Institute for Cancer and Allied Diseases, University of Nebraska Medical Center, Omaha, Nebraska, United States of America; 3 Department of Genetics, Osmania University, Hyderabad, Andhra Pradesh, India; 4 Cell Signaling Laboratory, Center for Cancer and Metabolism, Bioscience Division, SRI International, Menlo Park, California, United States of America; 5 Cell Signaling and Tumor Angiogenesis Laboratory, Department of Genetics, Boys Town National Research Hospital, Omaha, Nebraska, United States of America; Ohio State University, United States of America

## Abstract

**Object:**

Antiangiogenic treatments are beginning to give promising outcomes in many vascular diseases including tumor angiogenesis. In this current study the antiangiogenic and pro-apoptotic actions of α1(IV)NC1 and its N- and C- peptides α1S1(IV)NC1, α1S2(IV)NC1 were investigated *in-vitro* and *in-vivo*.

**Study Method:**

Endothelial cells (ECs) were treated with α1(IV)NC1, α1S1(IV)NC1, α1S2(IV)NC1 and *in-vitro* proliferation, migration, tube formation and apoptotic assays were executed. FasL, Fas, Caspase-8, -3 and PARP activations were studied using immunoblotting analysis using specific antibodies. Also the *in-vivo* antiangiogenic and pro-apoptotic effects were tested using α1(IV)NC1 in a mice model.

**Results:**

Like α1(IV)NC1, its N- and C- terminal α1S2(IV)NC1 and α1S1(IV)NC1 domains posses anti-proliferative, pro-apoptotic activity and inhibit ECs migration and tube formation *in-vitro*. Both α1S1(IV)NC1 and α1S2(IV)NC1 domains promote apoptosis by activating FasL and down stream apoptotic events including activation of caspase-8, -3 and PARP cleavage in a dose dependent manner *in-vitro* in ECs. Tumors in mice showed apoptotic TUNEL positive microvasculature upon α1(IV)NC1 treatment, indicating inhibition of tumor angiogenesis and tumor growth. Further, the antitumor activity of α1(IV)NC1 was abrogated when caspase-3 inhibitor was used. These results conform additional properties of α1(IV)NC1 as an endogenous angioinhibitor that induces apoptosis *in-vitro* and *in-vivo* by activating FasL mediated caspase-3.

**Significance:**

α1(IV)NC1 and its N- and C- terminal α1S1(IV)NC1 and α1S2(IV)NC1 domains also posses pro-apoptotic and angioinhibitory activity *in-vitro and in-vivo*. α1(IV)NC1 regulates tumor angiogenesis by activating FasL mediated apoptosis *in-vitro* and *in-vivo*. These results demonstrate that α1(IV)NC1 and its peptides inhibit neo-vascular diseases.

## Introduction

Angiogenesis, the formation of new blood vessels from preexisting blood vessels, is a very stringently controlled program and normally does not occur, except during development and wound repair processes [1], [2]. This stringent regulation of angiogenesis is manifested by a balance between pro-and anti-angiogenic factors, which keep angiogenesis in check [2]. However, the dynamic equilibrium between pro-angiogenic and anti-angiogenic factors are controlled under many pathological settings, including tumor angiogenesis in cancer progression and other incidents like as age-related macular degeneration, retinopathy of prematurity and diabetic retinopathy resulting in the growth of abnormal new blood vessels [3]–[5].

Vascular basement membranes (VBM) constitute an important component of blood vessels [6]. Makeover of VBM can provide vital pro- and anti-angiogenic molecules to control formation of new blood vessels [7]–[9]. Type IV collagen is a major component of VBM and plays a critical role in new blood vessel development [6]. Proteolytic degradation of type IV collagen in the VBM generates numerous antiangiogenic molecules [7], [10]–[12]. One such antiangiogenic molecule derived from type IV collagen non-collagenous (NC1) domain α1 chain, α1(IV)NC1, has been tested in variety of tumor angiogenesis studies in mice [13]–[15]. However, the molecular and cellular mechanism(s) responsible for inhibition of angiogenesis is not yet clearly understood. The *in-vitro* and *in-vivo* studies have demonstrated that α1(IV)NC1 can directly affect endothelial cell migration and impact their proliferation and sprouting [14]. Earlier we have demonstrated that α1(IV)NC1 promotes apoptosis via activation of caspase-3 and PARP cleavage by inhibiting FAK/p38-MAPK/Bcl-2 and Bcl-x**_L_** signaling cascade [15]. These results provide a clear understanding about the apoptotic signaling and therapeutic potential of α1(IV)NC1 molecule in neovascular diseases. However, the effects of α1(IV)NC1 and its N- and C-terminal domains α1S1(IV)NC1 and α1S2(IV)NC1 on endothelial cell apoptosis and neo-vascularization have not been previously studied.

In the present study, we demonstrate that α1(IV)NC1 and its N- and C-terminal domains α1S1(IV)NC1 and α1S2(IV)NC1 are potent inhibitors of endothelial cell proliferation, migration and tube formation *in-vitro* and tumor angiogenesis *in-vivo*. α1(IV)NC1 promotes apoptosis via activation of caspase-3 and PARP cleavage, presumably by inhibiting FAK/p38-MAPK/Bcl-2 and Bcl-x**_L_** signaling cascade [15]. Here in this study, we show that N- and C-terminal domains of α1(IV)NC1 cross talk with FasL and activate FasL and its downstream apoptotic missionary including caspase-8, caspase-3 and PARP cleavage *in-vitro*. Furthermore, we identified that α1(IV)NC1 promotes apoptosis in tumor vasculature and inhibits angiogenesis and this effect was reversed by a caspase-3 specific inhibitor DEVD *in-vivo*. These findings contribute significantly towards understanding the apoptotic activation in proliferating ECs and therapeutic potential of endogenous angioinhibitor α1(IV)NC1 and its N- and C-terminal α1S1(IV)NC1 and α1S2(IV)NC1 domains in tumor growth and tumor angiogenesis.

## Materials and Methods

Fetal calf serum (FCS), Endothelial basal medium (EBM-2) and Endothelial cell growth medium (EGM-2) were obtained from Fischer Scientific Inc. Penicillin and streptomycin and low melting agarose were purchased from Sigma-Aldrich and cell stains hematoxylin and eosin (H&E) were purchased from Fischer Scientific Inc. Sephadex™-G 100, -G 25 and -G 200 were purchased from GE Healthcare Bio-Sciences AB. BD Matrigel™ Matrix (14.6 mg/ml) was purchased from BD Biosciences Discovery Laboratory. T_4_-DNA ligase (bacteriophage ligase), different restriction enzymes and polymerases were purchased from New England Biolabs. SCC-PSA1 tumor cells were purchased from ATCC. Caspase inhibitors z-DEVD-fmk was from Enzyme System Products. H&E staining kit form Fisher Scientific Inc. CD31 antibody was purchased from Upstate. Caspase-3, -8, PARP, FasL and Fas antibodies were purchased from Cell Signaling. MTT assay kit was purchased from Chemicon.

### 

#### Ethics Statement

Boys Town National Research Hospital IACUC committee approved all mice studies.

### Tissue culture procedure

SCC-PSA1 cells were maintained in 10% FCS with penicillin/streptomycin (100units/ml each) and maintained at 37°C incubator with 5% CO_2_. Primary mouse choroidal endothelial cells (EC) were maintained as describe previously [16], [17]. EC were maintained in 40% HAM's F-12, 40% DME-Low Glucose, 20% FCS supplemented with heparin (50 mg/L), endothelial mitogen (50 mg/L), L-glutamine (2.0 mM), penicillin/streptomycin (100units/ml each), Na Pyruvate (2.5 mM), NEAA (1X), 5.0 µg/L of murine INF-γ and cultured on 0.8% gelatin coated plates at 33°C with 5% CO_2_.

### Cloning, Expression and Purification of α1(IV)NC1 and its N- and C- terminal domains α1S1(IV)NC1 and α1S2(IV)NC1

The coding sequence corresponding to the N- and C-terminal non-collagenous domains (NC1) from human Collagen type IV α1 chain was isolated from the placental cDNA using one-step reverse transcriptase-PCR (RT-PCR) (Invitrogen, CA). The N- terminal subunit 330-bp from full length α1(IV)NC1 was amplified using the forward primer: ATACATATGGGCTTCCTTGTGACCAGGCATA and the reverse primer: CACAAGCTTAGGCGCCTCACACACAGCAC and cloned between ‘NdeI’ and ‘XhoI’ sites of pET22b. The C- terminal subunit 330-bp from full length α1(IV)NC1 was amplified using the forward primers: GTGCATATGGTGCACAGCCAGACCAT and the reverse primer: GTGGCAGCAGCCAACTCA and cloned between ‘NdeI’ and ‘XhoI’ sites of pET22b. Amplification and cloning was carryout similarly as reported in our earlier publication [18]. The positive clone was used to transform *E. coli* strain BL21 for protein expression and purification was performed similarly as reported earlier [18].

### Purification of α1(IV)NC1 and α1S1(IV)NC1 and α1S2(IV)NC1 domains

Inclusion bodies of α1(IV)NC1, α1S1(IV)NC1 and α1S2(IV)NC1 were prepared with minor modifications as reported [18]. In addition to the renaturation by stirring method, on-column renaturation was performed for simultaneous renaturation and purification of the α1(IV)NC1, α1S1(IV)NC1 and α1S2(IV)NC1 domains. Denatured α1(IV)NC1, α1S1(IV)NC1 and α1S2(IV)NC1 protein in 800 µl aliquots was loaded onto the Sephadex G-100, Superdex-200 followed by Sephadex G-25 columns similarly as reported [18]. The fractions containing α1(IV)NC1, α1S1(IV)NC1 and α1S2(IV)NC1 were pooled and further concentrated by lyophilization as reported. Endotoxin levels in the final purified α1(IV)NC1, α1S1(IV)NC1 and α1S2(IV)NC1 domains samples were estimated using the Limulus Amebocyte Lysate (LAL) QCL-1000 assay kit (Lonza) according to the manufacturer instructions and also similarly reported in earlier publication [18].

### Proliferation assay

A suspension of 7000-cells/well mouse choroidal endothelial cells (ECs) in a 96 well plate was used in proliferation assay. Cells were grown in 96 well plate under 0.5% FBS supplemented with heparin, endothelial mitogen, glutamine and penicillin/streptomycin. After 24-hrs, medium was replaced with ECs medium containing 10% FCS and different concentrations of α1(IV)NC1 or its N- and C-terminal α1S1(IV)NC1 and α1S2(IV)NC1 domains (0.25 and 2.0 µM) and after 48-hrs relative levels of methylene blue incorporation was measured as reported [14], [17].

### Migration assay

About 1.0×10^4^ cells/well of ECs were seeded in serum free medium with and without recombinant α1S1(IV)NC1 and α1S2(IV)NC1 (1.0 µM). Medium containing 10 ηg/ml of VEGF was placed into the bottom wells of the Boyden chamber and incubated for about 48-hrs at 37°C with 5% CO_2._ The numbers of ECs that were migrated and attached to the bottom side of the Boyden chamber membrane were counted as reported earlier [14], [19].

### Tube formation assay

Briefly, Matrigel matrix about 250 µl was thawed overnight on ice-cold room and added to each well of a 24-well plate and allowed to solidify for 30-min at 37°C culture incubator. A suspension of about 50×10^3^ ECs in medium without antibiotic was plated on top of the Matrigel matrix. The ECs were than incubated with and without α1(IV)NC1 or its N- and C-terminal α1S1(IV)NC1 and α1S2(IV)NC1 domains (1.0 µM) for 48-hrs at 37°C and viewed using a CK2 Olympus microscope [14], [20], [21].

### Cell viability assay

Endothelial cell viability was assessed by MTT assay [3-(4,5-dimethylthiazol-2-yl)-2,5-diphenyl tetrazoliumbromide] following the manufacturer's protocol instructions. About 7.0×10^3^ EC cells/well were plated on a 96-well plate, over night serum starved and stimulated with 10% FCS containing medium. After 24-hrs different concentration of α1(IV)NC1 or its N- and C-terminal α1S1(IV)NC1 and α1S2(IV)NC1 domains (0.25–2.0 µM) were added and incubated for 48-hrs. Apoptosis was monitored by trypan blue exclusion using the cell death detection ELISA kit [22].

### FasL and caspase activation assay

About 1.0×10^6^ serum starved EC were collected and suspended in serum free medium. These ECs were pretreated with α1(IV)NC1 N- and C-terminal α1S1(IV)NC1 and α1S2(IV)NC1 domains (1.0 µM) and incubated on 10-cm^2^ dishes for 6 and 18-hrs. After 6 and 18-hrs, the floating ECs were collected, adherent cells washed once with cold PBS. Adhering and floating cells were lysed on ice in RIPA lysis buffer and centrifuged at 4°C for 30-min at 13000-rpm. About 30 µg of cytosolic extract/lane was separated using 10% SDS-PAGE followed by immunoblotting with anti-FasL, Fas, caspase-8, caspase-3 and PARP antibodies. Immunoreactivity of FasL, Fas, cleaved caspase-8, caspase-3 and PARP proteins were visualized using ECL detection kit as reported [15].

### *In-vivo* tumor studies

About 1.0×10^6^ SCC-PSA1 teratocarcinoma tumor cells were implanted subcutaneously into each mouse at dorso-lateral sides. Six male 129/Sv mice were used in each treatment and control groups. Once the tumors reached around 100-mm^3^ sizes, 30 µg of α1(IV)NC1 or in combination with 10 µg of caspase-3 specific inhibitor DEVD in 100 µl total volume was intravenously injected to each mouse daily for 15-days. All the experimental mice were scarified and tumors and other organs were collected [21], [31].

### Immunohistochemical staining

Tumors from control and α1(IV)NC1 or in combination with DEVD treated mice were embedded in OTC compound and snap-frozen in liquid nitrogen. Tumor sections were incubated for 2-hrs with 5% BSA in PBS to block any nonspecific binding and then incubated with anti-CD-31 antibody for 1-hr followed by incubation with peroxidase-labeled goat anti-rabbit secondary antibody at 37°C. The number of CD-31 positive blood vessels per 10-microscopic fields from four tumor sections per condition was counted [14], [23].

### TUNEL assay for apoptosis

To evaluate the relative levels of CD-31 positive vascular or cellular apoptosis, and to quantify cellular apoptosis in tumors from control and α1(IV)NC1 or in combination with DEVD treated mice with and without α1(IV)NC1 treatment, we used ApopTag apoptosis detection kit. Briefly the tumor sections were incubated with 5% BSA in PBS to block any nonspecific binding and stained with ApopTag apoptosis detection reagents following the manufacturer's instructions. To determine whether CD-31 positive blood vessels were undergoing apoptosis, control and α1(IV)NC1 or in combination with DEVD treated tumor sections were co-incubated with both ApopTag reagents and anti-CD-31 antibody. Tumor sections were washed twice and incubated with rhodamine-conjugated goat anti-rabbit secondary antibody for 1-hr at 37°C. The fluorescence staining was analyzed using a fluorescence microscope [14], [23].

## Results

### Distinct anti-angiogenic activities of α1(IV)NC1 and its N- and C- terminal domains α1S1(IV)NC1 and α1S2(IV)NC1

α1(IV)NC1 is an endogenous metabolite generated from the non-collagenous (NC1) domain of α1 chain of type IV collagen by matrix metalloproteinases-9 (MMP-9) [24], [25]. It was discovered as an angioinhibitory protein with substantial anti-tumor activities [13], [14]. The present study was aimed at understanding the molecular mechanism(s) underlying angioinhibition by α1(IV)NC1 and its N- and C- terminal α1S1(IV)NC1, α1S2(IV)NC1 domains and its implications in prevention of tumor angiogenesis. Our studies tried to identify the angioinhibitory and pro-apoptotic activities of α1(IV)NC1 and its N- and C- terminal α1S1(IV)NC1, α1S2(IV)NC1 domains in mouse choroidal endothelial cells (ECs).

**Figure 1:**
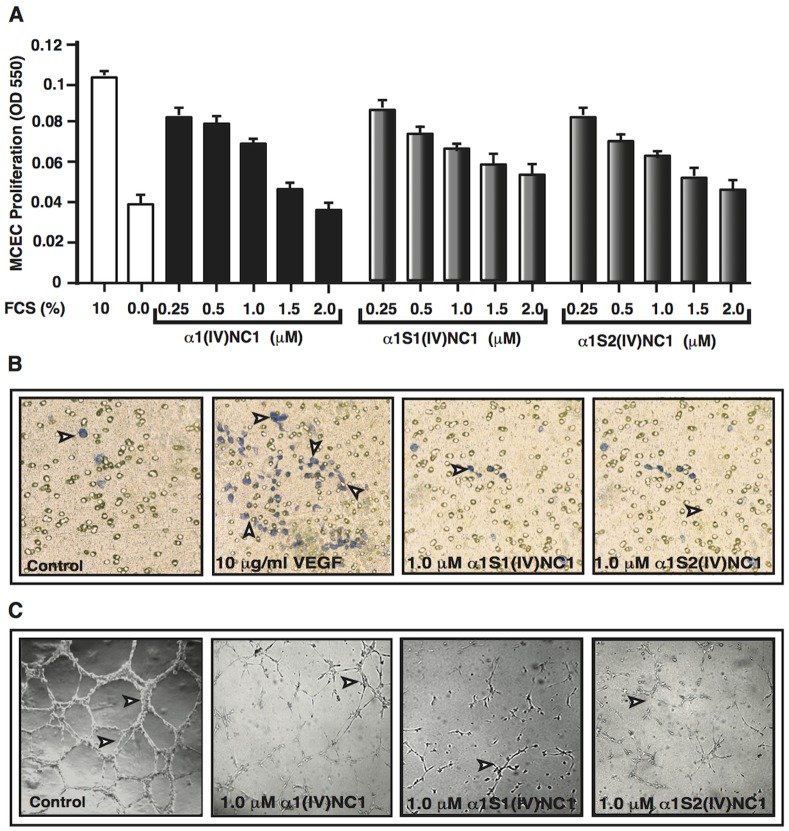
Proliferation (A). Graph summarizes relative levels of methylene blue incorporation in mouse choroidal endothelial cells (ECs) treating with different concentrations of α1(IV)NC1 and its N- and C terminal α1S1(IV)NC1 and α1S2(IV)NC1 domains compared with and without FCS controls after 48-hrs. All groups represent triplicate samples and data in the graphs are represented as mean±SD. **Migration (B)**. Number of ECs with and without α1S1(IV)NC1 and α1S2(IV)NC1 domains migrated towards VEGF on the underside of Boyden chamber membrane were shown and cells viewed using a light microscope after 48-hrs representative fields (100× magnification) shown. **Tube formation (C)**. ECs were plated on Matrigel coated plates in endothelial cell medium as control or with 1.0 µM α1(IV)NC1, α1S1(IV)NC1 and α1S2(IV)NC1 proteins. Tube formation was assessed using a light microscope after 48-hrs, and representative fields at 100× magnification were shown.

We have performed different angiogenesis experiments to determine the angioinhibitory potential of α1(IV)NC1 and its N- and C-terminal domains α1S1(IV)NC1 and α1S2(IV)NC1 using ECs. Many serum proteins are major pro-angiopromoting factors, elevated levels or imbalance of serum proteins (example VEGF and bFGF) are responsible for the majority of ocular angiogenesis driven by ischemia and also tumor angiogenesis. We first determined serum stimulated angioinhibitory activity of α1(IV)NC1 and its N- and C-terminal domains α1S1(IV)NC1 and α1S2(IV)NC1 by measuring ECs proliferation. The anti-proliferative effect of α1(IV)NC1 and its N- and C-terminal domains were tested in ECs using methylene blue incorporation. Fetal calf serum (10% FCS) stimulated proliferation of ECs were significantly inhibited by α1(IV)NC1 its N-and C-terminal α1S1(IV)NC1 and α1S2(IV)NC1 domains in a dose dependent manner (**Figure 1A**). Interestingly, both N- and C-terminal α1S1(IV)NC1 and α1S2(IV)NC1 domains were showing similar proliferation inhibitory activity when compared to its parent molecule α1(IV)NC1.

Migration of endothelial cells is basically essential during neo-vascularization [14], [26]. Migration of ECs across a PVD membrane towards VEGF in a Boyden chamber was inhibited by 1.0 µM N-and C-terminal α1S1(IV)NC1 and α1S2(IV)NC1 domains (**Figure 1B**). We additionally confirmed the angioinhibitory action of α1(IV)NC1 and its two domains α1S1(IV)NC1 and α1S2(IV)NC1 by a functional assay of ECs tube formation [20]. Tube formation on Matrigel is associated with ECs proliferation, migration and survival [1]. Treatment of ECs with α1(IV)NC1 and its domains α1S1(IV)NC1 and α1S2(IV)NC1 inhibited tube formation equally on Matrigel matrix (**Figure 1C**). Our previous study reported that the angioinhibitory activity of α1(IV)NC1 is mediated through α1β1 integrin [14], [21]. Surprisingly, in this study we noticed that ECs when treated with N- and C-terminal domains and full length α1(IV)NC1 (1.0 µM) were appeared rounded (apoptosis-like) and some of the cells detach from the Matrigel matrix **(data not shown)**. This might be due to activation caspases and apoptosis in ECs treated with α1(IV)NC1 or its N- and C- terminal domains α1S1(IV)NC1 and α1S2(IV)NC1 similarly as reported earlier [15].

**Figure 2:**
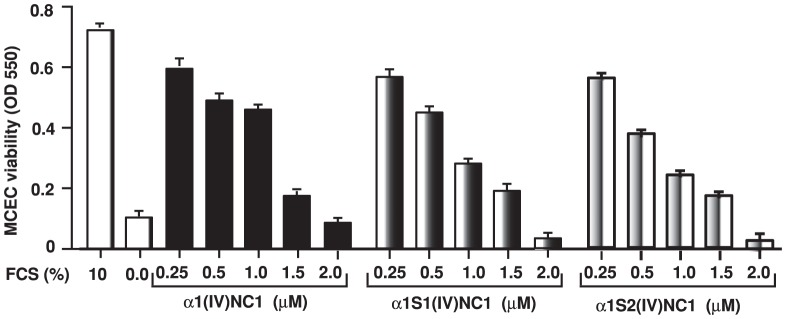
Cell viability. MTT assay was performed to evaluate mouse choroidal endothelial cells (ECs) viability after treatment with different concentrations of α1(IV)NC1 and its N- and C- terminal domains α1S1(IV)NC1 and α1S2(IV)NC1. ECs grown with and without FCS as positive and negative controls. Experiments were performed with three replicates and data in the graphs are represented as mean±SD.

Further we also tested the angioinhibitory activity at different doses of α1(IV)NC1 and its N-and C- terminal domains α1S1(IV)NC1 and α1S2(IV)NC1 (0, 0.25, 0.5, 1.0, 1.5, 2.0 µM) by MTT cell viability assay after 10% FCS stimulation in ECs. The results reveal that ECs proliferation was significantly increased by FCS stimulation which was inhibited by α1(IV)NC1 and its N- and C-terminal domains dose dependently after 24-hrs (**Figure 2**).

### FasL mediated *in-vitro* apoptotic activity of α1(IV)NC1 and its N- and C- terminal α1S1(IV)NC1 and α1S2(IV)NC1 domains

**Figure 3:**
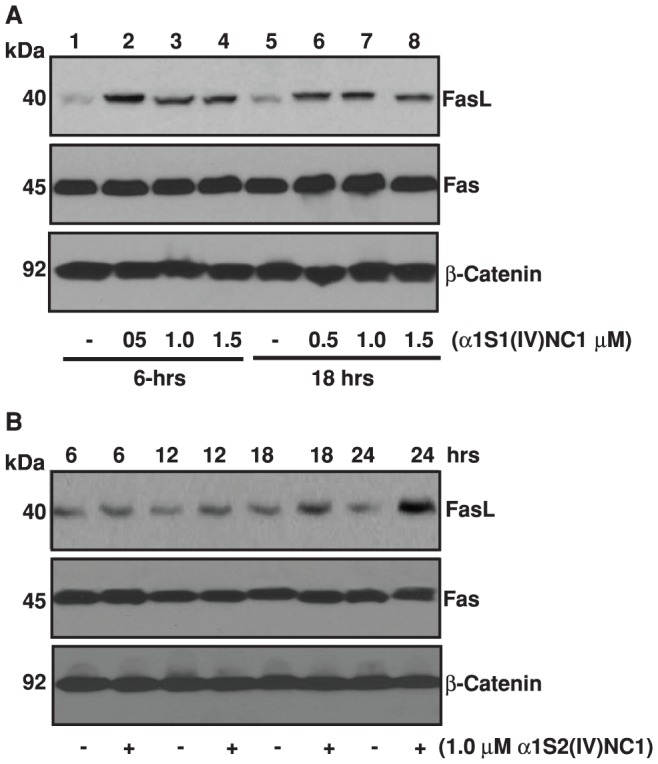
FasL activation (A and B). Mouse choroidal endothelial cells were incubated with and without different doses of α1S1(IV)NC1 and α1S2(IV)NC1 domains for 6 and 18-hrs, and total cells were collected, lysed for 30-min in ice-cold RIPA lysis buffer and about 25 µg of total cytosolic extract per lane was separated and immunoblotted with primary antibodies against FasL and Fas. In panel A and B, β-catenin was shown as loading control.

Caspase-3 is a pivotal molecule mediating cellular apoptosis [27]. Earlier we demonstrated that α1(IV)NC1 induces apoptosis in endothelial cells by activating Caspase-3 [15]. Here in this study, we tested, whether α1(IV)NC1 and its N- and C-terminal domains posses pro-apoptotic activity or not? Interestingly, ECs incubated with α1(IV)NC1 and its N- and C- terminal domains showed dose and time dependent activation of FasL without affecting Fas expression compared to control untreated cells (**Figure 3A & B**). These results suggest both α1S1(IV)NC1 and α1S2(IV)NC1 domains may cross talk with death receptor, activating FasL mediated apoptosis in ECs. These results demonstrate that α1(IV)NC1 and its two domains α1S1(IV)NC1 and α1S2(IV)NC1 promoted apoptosis through FasL in addition to MAPK signaling inhibition [**15**].

**Figure 4:**
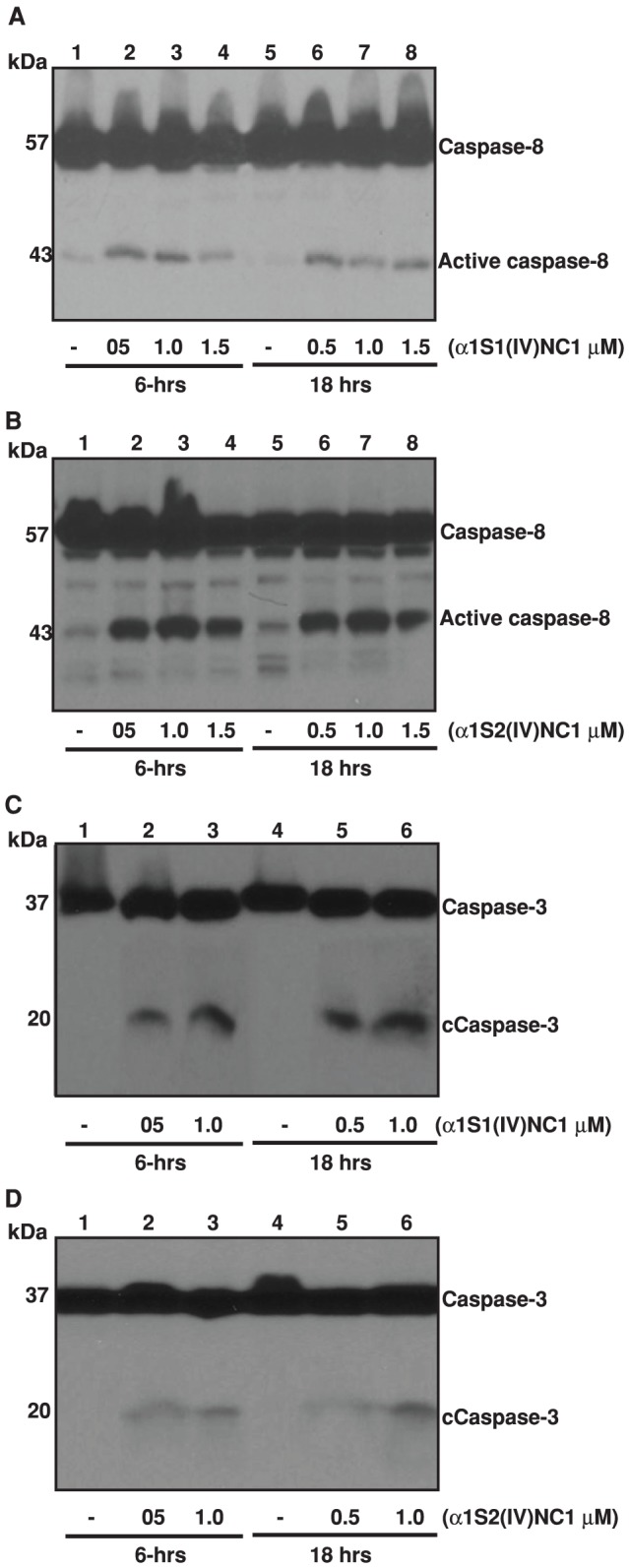
Caspase-8 activation (A and B). Mouse choroidal endothelial cells (ECs) were incubated with and without different doses of α1S1(IV)NC1 and α1S2(IV)NC1 domains for 6 and 18-hrs, and total cells lysed for 30-min in ice-cold RIPA lysis buffer and about 25 µg of cytosolic extract per lane was separated and immunoblotted with primary antibodies against caspase-8. **Caspase-3 activation (C and D)**. ECs were incubated with and without different doses of α1S1(IV)NC1 and α1S2(IV)NC1 domains and total cytosolic extract immunoblotted with primary antibodies against caspase-3.

**Figure 5:**
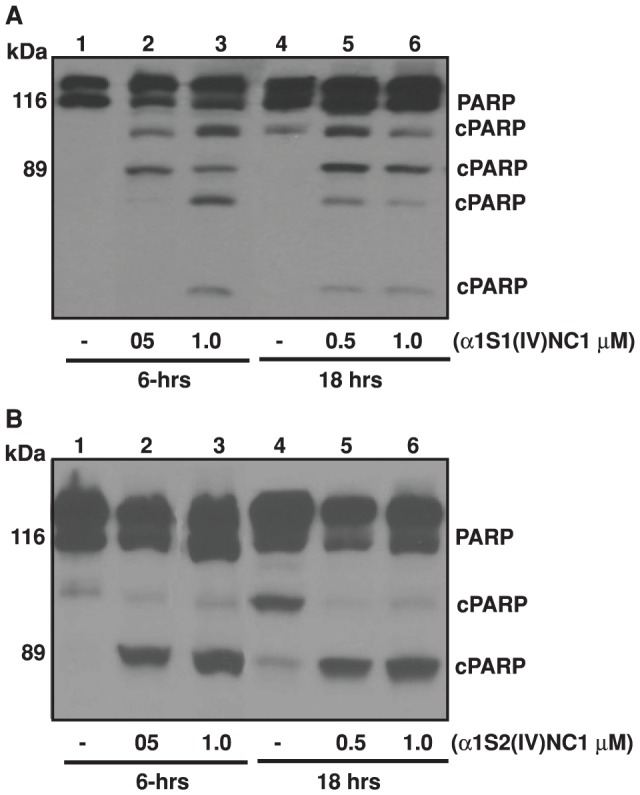
PARP activation (A and B). Mouse choroidal endothelial cells (ECs) were incubated with and without different doses of α1S1(IV)NC1 and α1S2(IV)NC1 domains for 6 and 18-hrs, and total cells lysed for 30-min in ice-cold RIPA lysis buffer and about 25 µg of cytosolic extract per lane was separated and immunoblotted with primary antibodies against PARP.

We further assessed whether α1S1(IV)NC1 and α1S2(IV)NC1 induces activation of FasL downstream different caspases such as caspase-8 and caspase-3. We treated ECs with α1S1(IV)NC1 and α1S2(IV)NC1 and observed that activation of caspase-8 (**Figure 4A and B**). These results demonstrate that full length α1(IV)NC1, both its N- and C- terminal domains α1S1(IV)NC1 and α1S2(IV)NC1 induced cellular apoptosis by activating FasL and activating downstream caspase-8. Among the known caspases, caspase-3 is an important effector molecule for most cellular apoptosis [27]. To study whether caspase-3 could be activated by α1S1(IV)NC1 and α1S2(IV)NC1 domains, we treated ECs with different doses of α1S1(IV)NC1 and α1S2(IV)NC1 and observed activation of caspase-3 in a time dependent manner (**Figure 4C and D**). Here, we also identified that activation PARP cleavage which is further down stream to FasL, caspase-8 and caspase-3 in ECs treated with α1S1(IV)NC1 and α1S2(IV)NC1 time and dose dependently (**Figure 5A and B**). These results demonstrate that activation of ECs apoptosis by α1S1(IV)NC1 and α1S2(IV)NC1 domains by activating FasL and its down stream caspases and PARP cleavage.

### Neutralizing anti-tumorigenic activity of α1(IV)NC1 by caspase-3 inhibitor

To further conform the pro-apoptotic activity of α1(IV)NC1 also partly regulated by caspase-3 activation and apoptosis, we carried *in-vitro* tumor studies with α1(IV)NC1, or α1(IV)NC1 co-administrated with caspase-3 inhibitor DEVD to tumor bearing mice. Consistent to our earlier findings, administration of α1(IV)NC1 significantly inhibited SCC-PSA1 tumor growth and tumor angiogenesis *in-vivo* [27]. In contrast, treatment with caspase-3 specific inhibitor DEVD alone showed significant effect on inhibition of tumor growth. The antitumor activity of α1(IV)NC1 was significantly attenuated when α1(IV)NC1 and DEVD were co-administrated to tumor bearing mice (data not shown).

**Figure 6:**
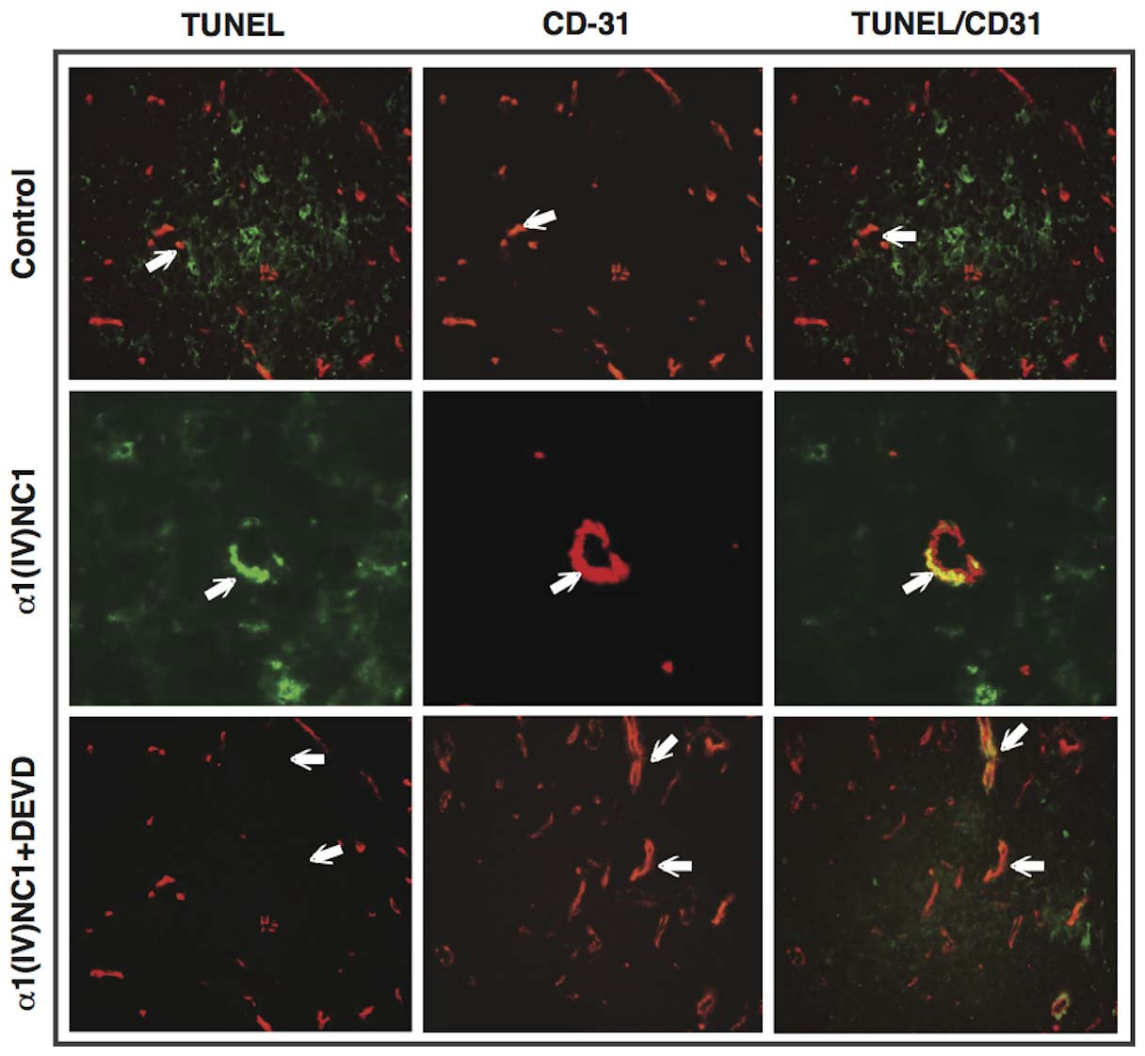
Attenuation of antitumor activity and tumor vasculature apoptosis by caspase-3 inhibitor. α1(IV)NC1 alone or together with DEVD was injected into SCC-PSA1 tumor bearing mice daily for 15-days. Frozen sections (4.0 µm) of tumors were examined through immunohistochemistry. Apoptotic TUNEL positive cells (green, left), and CD-31 positive tumor blood vessel counts were scored from 10-microscopic fields and four tumors for each experimental condition (red, middle) and co-localization (yellow, right) was shown at 100× magnification. Scale bar corresponds to 50 µm.

To assess the antitumor effects of α1(IV)NC1 and its ability to activate microvasculature apoptosis, control and α1(IV)NC1 treated mice tumor sections were stained with anti-CD31 antibody (**Fig. 6** **, middle panel**). In control tumors, less TUNEL positive apoptotic staining was detected whereas elevated levels of apoptosis was observed in tumors that were treated with α1(IV)NC1 (**Fig. 6**). DEVD treatment alone meagerly affected overall tumor cell apoptosis when compared with α1(IV)NC1. Further, a significant number of ECs in tumor vasculature underwent apoptosis upon α1(IV)NC1 treatment when compared to control tumors, as observed through CD31/TUNEL dual staining (**Fig. 6** **, right panel yellow**). Interestingly, co-administration of α1(IV)NC1 with DEVD to tumor bearing mice significantly reduced EC apoptosis in microvasculature that was induced by α1(IV)NC1. Further apoptotic tumor cells were observed in α1(IV)NC1 treated tumors using TUNEL staining. This could be the result of an indirect effect since the microvasculature is affected by α1(IV)NC1 treatment, and the tumor cells possibly suffer from oxygen supply and nourishment resulting in apoptosis. This effect was observed at elevated levels in cells undergoing apoptosis, other than CD-31 positive ECs (**Fig. 6** **, left and right panels**). We also quantified the microvascular density and number of apoptotic vessels through TUNEL staining and by counting the number of CD-31 positive blood vessels (data not shown). Collectively, our results demonstrate that α1(IV)NC1 and its -N and -C terminal domains induced activation of FasL mediated caspase-3 activation and apoptosis in proliferating endothelial cells.

## Discussion

Many endogenous metabolites generated from type IV collagen were identified as pro-apoptotic, antiangiogenic and anti-tumorigenic in nature [12]–[15], [21], [25], [28]–[32]. The non-collagenous (NC1) domain released by proteolytic activity from type IV collagen α1 chain (α1(IV)NC1) was identified as an antiangiogenic molecule, where as its N- and C terminal domains apoptotic activity is not yet studied. Antiangiogenic activity of α1(IV)NC1 is mediating through α1β1 integrin [14], [21]. However, it is critical to examine α1(IV)NC1 and its both its N- and C- terminal domains α1S1(IV)NC1 and α1S2(IV)NC1 effects in several well defined relevant *in-vitro* experiments before confirming that these domains are also antiangiogenic. In this study, we tested the antiangiogenic/pro-apoptotic effects of α1(IV)NC1 and its N- and C- terminal domains in different *in-vitro* and *in-vivo* experiments.

We demonstrate in this study for the first time, that α1(IV)NC1 and its N- and C- terminal domains inhibits serum induced EC proliferation, migration and tube formation. In addition, we also identified that both α1S1(IV)NC1 and α1S2(IV)NC1 domains activates caspase-8, caspase-3/PARP cleavage through FasL activation in proliferating endothelial cells. This is coherent with the earlier studies revealing that antiangiogenic activity of α1(IV)NC1 is mediated through α1β1 integrin signaling and apoptosis [14], [33]. α1(IV)NC1 and its N- and C- terminal α1S1(IV)NC1 and α1S2(IV)NC1 domains activated FasL mediated apoptosis in ECs. Understanding the mechanism(s) of action of α1S1(IV)NC1 and α1S2(IV)NC domains is crucial for their therapeutic development and use. Thus, α1(IV)NC1 and its N- and C- terminal domains promotes apoptosis in proliferating ECs and inhibits tumor angiogenesis, these endogenous molecules may be an effective therapeutic candidate for treatment of many neovascular diseases. Further evaluation through extensive laboratory studies on these molecules is needed to address the function of these angioinhibitors to be considered for the clinical trials.

Earlier lessons from preclinical trials of angiostatin, endostatin, Thrombospondin-1 (ABT-510) and 2-ME suggest that more basic laboratory studies are required to better understand the mechanism of actions associated with these angioinhibitor molecules. Presently, some of the angioinhibitory agents such as Bevacizumab and VEGFR tyrosine kinase inhibitors; Vatalanib (PTK787/ZK 222584), Semaxanib (SU5416), Sunitinib (SU11248), Sorafenib (BAY 43-9006) are in clinical trials [34], [35]. Many other angioinhibitory drugs [Macugen (pegaptanib sodium), Lucentis (formerly RhuFab V2), tryptophanyl-tRNA synthetase (TrpRS), VEGF-TRAP, AdPEDF, AG-013958, Avastin (bevacizumab), JSM6427 etc] inhibit ocular neovascularization and prevent leakiness of retinal blood vessels by preventing binding of VEGF to its receptors on endothelial cells [15], [36]–[42]. Endogenous metabolite, α2(IV)NC1/α3(IV)NC1 were also reported to laser induced CNV by promoting apoptosis of endothelial cells *in-vivo* [10].

Our findings suggest that α1(IV)NC1 and its N- and C- terminal domains α1S1(IV)NC1 and α1S2(IV)NC1 may also be effective in inhibition of tumor angiogenesis by activating caspase-3 in new blood vessels. This is further supported by earlier findings that another angioinhibitor, α3(IV)NC1 regulate angiogenesis in a number of *in-vitro* and *in-vivo* models [10]. Regression of tumor growth in mice upon α1(IV)NC1 treatment is associated with reduced tumor vasculature and increased TUNEL positive endothelial and tumor cells when compared to control tumors, indicating *in-vitro* activation of caspase-3. This suggests that α1(IV)NC1 activates caspase-3 and inhibits tumor angiogenesis and tumor growth. Thus, this work not only supports our efforts in development of α1(IV)NC1, its N- and C- terminal domains α1S1(IV)NC1 and α1S2(IV)NC as a potential candidate for tumor angiogenesis neovascular diseases.
